# Supplementary material for: End Sequence Analysis Toolkit (ESAT) expands the extractable information from single-cell RNA-seq data
Source: Genome Res. 2016 Oct;26(10):1397–410. doi: 10.1101/gr.207902.116 (PMC5052061; doi:10.1101/gr.207902.116)
Supplement: Supplemental Material [file supp_gr.207902.116_Supp_Materials.tar.gz › ESAT-esat_v0.1/doc/html/classbroad_1_1core_1_1parser_1_1_string_parser-members.html]

ESAT: Member List


ESAT


- Main Page
- Related Pages
- Packages
- Classes
- Files

- Class List
- Class Index
- Class Hierarchy
- Class Members

All Classes Namespaces Files Functions Variables Enumerator Pages

- broad
- core
- parser
- StringParser

broad.core.parser.StringParser Member List

This is the complete list of members for broad.core.parser.StringParser, including all inherited members.

|  |  |  |
| --- | --- | --- |
| asDouble(int index) | broad.core.parser.StringParser |  |
| asFloat(int index) | broad.core.parser.StringParser |  |
| asInt(int index) | broad.core.parser.StringParser |  |
| asLong(int index) | broad.core.parser.StringParser |  |
| asString(int index) | broad.core.parser.StringParser |  |
| clear() | broad.core.parser.StringParser |  |
| firstField(String s) | broad.core.parser.StringParser | static |
| getFieldCount() | broad.core.parser.StringParser |  |
| getIndexFor(String string) | broad.core.parser.StringParser |  |
| getStringArray() | broad.core.parser.StringParser |  |
| getTokens(String s) | broad.core.parser.StringParser | static |
| parse(String s) | broad.core.parser.StringParser |  |
| parse(String s, String regexp) | broad.core.parser.StringParser |  |
| StringParser() | broad.core.parser.StringParser |  |


---

Generated on Fri Aug 15 2014 15:10:55 for ESAT by  

 1.8.7
